# The clinical and biological significance of STAT1 in esophageal squamous cell carcinoma

**DOI:** 10.1186/1471-2407-14-791

**Published:** 2014-10-29

**Authors:** Ying Zhang, Ommoleila Molavi, Min Su, Raymond Lai

**Affiliations:** Department of Pathology, Shantou University Medical College, 22 Xinling Road, Shantou, 515031 Guangdong Province China; Department of Laboratory Medicine and Pathology, University of Alberta, Edmonton, Alberta Canada; Department of Oncology, University of Alberta, Edmonton, Alberta Canada; DynaLIFEDX Medical Laboratories, Edmonton, Alberta Canada; Department of Laboratory Medicine and Pathology, University of Alberta, 5142 Katz Group Centre for Pharmacy and Health Research, Edmonton, Alberta T6G 2E1 Canada

**Keywords:** STAT1, Esophageal squamous cell carcinoma, Prognosis, NF-κB, STAT3

## Abstract

**Background:**

Loss of STAT1 (Signal Transducer and Activator of Transcription-1) has been implicated in the pathobiology of a number of cancer types. Nonetheless, the biological and clinical significance of STAT1 in esophageal squamous cell carcinomas (ESCC) has not been comprehensively studied.

**Methods:**

Using immunohistochemistry, we detected the STAT1 expression in a cohort of ESCC patients; *In-vitro* experiments, we used enforced gene transfection of *STAT1C* into two STAT1- weak/negative ESCC cell lines and siRNA knockdown of STAT1 in two STAT1-strong ESCC cell lines to detect STAT1 function in ESCC.

**Results:**

We found that the expression of STAT1 was heterogeneous in ESCC, with 64 (49.0%) strongly positive cases, 59 (45.0%) weakly positive cases and 8 (6.1%) negative cases. STAT1 expression inversely correlated with the depth of tumor invasion and tumor size (p=0.047 and p=0.029, respectively, Chi square). Furthermore, patients with STAT1-strong/weak tumors had a significantly longer survival compared to those with STAT1-negative tumors (33.6 months versus 13.1 months, p=0.019). In patients carrying tumors of aggressive cytology (n=50), those with STAT1-strong tumors survived significantly longer than those with STAT1-weak/negative tumors (34.6 months versus 20.5 months, p=0.011). Our *in-vitro* experiments revealed that STAT1 is proapoptotic and inhibitory to cell-cycle progression and colony formation. Lastly, we found evidence that STAT1 signaling in ESCC cells down-regulated the expression and/or activity of NF-κB and STAT3, both of which are known to have oncogenic potential.

**Conclusion:**

To conclude, our findings suggest that STAT1 is a tumor suppressor in ESCC. Loss of STAT1, which is frequent in ESCC, contributes to the pathogenesis of these tumors.

## Background

Members of the STAT protein family are known to regulate various cellular processes involved in oncogenesis, including cell cycle progression, apoptosis, angiogenesis, invasion, metastasis, and evasion of the immune system [1]. STAT1, as the first discovered member of the STAT family, serves as the principal mediator of both type I and type II interferon activation [2]. Recent studies have revealed that the expression of STAT1 is frequently lost in various types of human cancer such as breast cancer, head and neck cancer, multiple myeloma and leukemia [3]. Furthermore, it has been reported that STAT1 can inhibit the growth of benign and neoplastic cells by regulating the transcription and expression of a host of pro-apoptotic and anti-proliferative genes, such as caspases, BCL-xL and p21^waf1^ [4]. Overall, these observations suggest that STAT1 carries tumor suppressor functions.

Esophageal cancer is one of the leading causes of cancer-related deaths worldwide [5]. This type of cancer is known to be highly frequent in specific geographic regions in China, such as the Chaoshan area. Specifically, the annual average age-standardized incidence rate of esophageal cancer in Chaoshan is 74.5 /100,000 people [6], as compared to 7.0/100,000 people worldwide. This finding suggests that various genetic and/or environmental factors may predispose the population in Chaoshan to esophageal cancer. Interestingly, esophageal cancer found in Chaoshan predominantly carries the histology of squamous cell carcinoma, in contrast with that in the Western world which predominantly carries the histology of adenocarcinoma [7]. The pathogenesis of ESCC is incompletely understood. The overall survival of these patients remains to be relatively poor, with the overall 5-year survival rate being approximately 15% [8]. In one previous study, it was found that γ-interferon can induce significant apoptosis in ESCC cell lines and this process correlates with STAT1 activation [9]. In parallel with this observation, it was reported that the EGF-STAT1 signaling pathway, which is active in normal esophageal epithelial cells, is lost in a considerable fraction of esophageal cancer; furthermore, loss of EGF-STAT1 signaling was found to correlate with a worse clinical outcome [10]. Nevertheless, the clinical and biological significance of STAT1 in ESCC has never been directly or comprehensively examined.

In the present study, we tested our hypothesis that STAT1 is a tumor suppressor in ESCC. First, using immunohistochemistry and Western blots, we comprehensively evaluated the expression of STAT1 in a large cohort of ESCC harvested from patients from Chaoshan. Second, we evaluated the clinical and prognostic significance of STAT1 in ESCC. Third, we used an *in-vitro* model to assess the biological functions of STAT1 in ESCC cells.

## Methods

### ESCC tumor samples and cell lines

We collected 131 consecutive ESCC tumors at the Shantou Tumor Hospital between 2005 and 2012. All patients underwent potentially curative surgery without preoperative chemotherapy or radiotherapy. In this cohort, 98 were men and 33 were women; the age was 36-78 years, with a median of 57 years. Follow-up data was available for 74 patients; most (58, 78.4%) died during the follow-up period (median, 31.4 months). The study was approved by the ethical review committees of the Medical College of Shantou University. All participants involved in our study were given written informed consents.

Four ESCC cell lines (EC1, EC109, KYESE150 and KYSE510) and 4 human esophageal immortalized epithelial cell lines (SHEE, NE2, NE3, and NE6) were included in this study. The ESCC cell lines were gifts from Shantou University Medical College and esophageal immortalized epithelial cell lines were gifts from University of Hong Kong. All of them were cultured in DMEM supplemented with 10% fetal bovine serum at 37°C under 5% CO_2_.

### Antibodies, subcellular fractionation and western blotting

Western blot analysis was performed using standard techniques as previously described [11]. The following antibodies were employed: anti-STAT1 (1:1000) and anti-p-STAT1 (Tyr-701) (1:1000), anti-FLAG (1:1000), anti-caspase 3 (1:1000), anti-survivin (1:1000), anti- BCL-2 (1:1000) anti-p21 (1:1000) and anti-cyclin D1 (1:1000), all of which were purchased from Cell Signaling (Danvers, MA, USA). Anti-STAT3 (1:1000), anti-p-STAT3 (Tyr-705) (1:1000), anti-BCL-xL (1:1000) and anti-ß-actin (1:1000) were obtained from Santa Cruz Biotechnology (Santa Cruz, CA, USA). Densitometric analysis was performed using the ImageJ analysis system (Bethesda, WA, USA); the values for the STAT1 bands were normalized to those of the β-actin bands.

### Immunohistochemistry

Immunohistochemistry to detect STAT1 expression was performed using a method similar to that described previously [12]. Using the same antibody we employed for our Western blot studies, we performed immunohistochemistry and the staining results were independently evaluated by two pathologists who were blinded to the clinical data. For each case, the percentages of cells showing negative, weak or strong cytoplasmic STAT1 staining was recorded. Using our scoring system (the sum of % of cells strongly positive for STAT1 x 3 and % of cells weakly positive for STAT1 x 1), we determined that a cut-off of 80 points allowed us to achieve the lowest p-values in our statistical analysis. Thus, tumors with a score of <80 point were classified as STAT1-weak whereas those with a score of ≥80 points were classified as STAT1-strong.

### Co-immunoprecipitation

A total of 2 μg of anti-STAT3 monoclonal antibody (Santa Cruz Biotechnology) was added to 500 μg of protein lysate isolated in cell lytic M (Sigma Aldrich, St Louis, MD, USA) and the samples were rotated overnight at 4°C. Subsequently, 30 μl of protein G Plus/A beads (Emdmillipore, Billerica, MA, USA) was added to the samples and rocked overnight at 4°C. The beads were then washed 3 times with cold phosphate-buffered saline followed by the final wash using cold cell lysis buffer. Western blot analysis was then performed using standard techniques as previously described [11].

### Plasmids, cell transfection and NF-κB transcriptional activity

FLAG-tagged *STAT1C* cloned into the backbone of pcDNA3.1 was a gift from Dr. Ouchi (University of New York) [13]. For each experiment, 1 × 10^6^ ESCC cells were transiently transfected with 10 μg of *STAT1C* vector or the pcDNA3.1 empty vector (Invitrogen, Burlington, Ontario, CA) in 6-well plates using the lipofectamine 2000 reagent (Invitrogen) as per manufacturer’s suggested protocol. The NF-κB transcriptional activity analyses were performed as previously described [12].

### Short interfering RNA and gene transfection

5 × 10^6^ ESCC cells in 2 ml of culture medium were transfected with 100 pmol of SMARTpool-designed siRNA against STAT1 obtained from Dharmacon (Lafoyetle, CO, USA). Cells transfected with scrambled siRNA (Dharmacon) were used as the negative controls. Gene transfection was performed by using lipofectamine RNAiMax (Invitrogen) as per manufacturer’s suggested protocol.

### Cell-cycle analysis by flow cytometry and assessment of cell growth

Flow cytometry analyses were performed at the University of Alberta flow cytometry core facility as previously described [12]. All experiments were performed in triplicates.

To assess cell growth, ESCC cells were plated at a density of 20,000/ml of culture medium. Cell count, done daily for 4 days, was performed using trypan blue staining (Sigma-Aldrich) according to the manufacturer’s protocol. Triplicate experiments were performed.

### Colony formation assay

After *STAT1C* transfection, 500 cells/well were plated in six-well plates and incubated 10 days at 37°C. The cells were fixed with 4% buffered formalin for 15 min and then stained with 1% crystal violet (Sigma Aldrich) for 30 min. The plates were gently washed with PBS and dried before microscopic evaluation. Cell clusters with >30 cells were considered as a colony.

### Quantitative RT-PCR

Using the RNeasy Mini Kit (QINGEN, Valencia, CA, USA), total cellular RNA was extracted from cells following the manufacture’s protocol. Reverse transcription was performed using 1 μg of total RNA and superscript reverse transcriptase obtained from Invitrogen. Quantitative PCR was performed using SYBR green (Invitrogen), and the primer sets for STAT1 and GAPDH were purchased from Invitrogen. For both primer sets, the PCR conditions were as follows: 95°C for 10 minutes, followed by 40 cycles of 95°C for 15 seconds and 60°C for 1 minute. Samples were processed on an ABI 9700 HT system (Applied Biosystems Inc., Foster City, CA). Results were examined using the SDS 2.2 software, and the relative expression levels of STAT1 were calculated by normalizing with those of GAPDH.

### Cell invasion assay

The invasion assays were done using basement membrane (Cell biolabs, NY). The ESCC cell treat with *STAT1C* or empty vector were prepared before the experiment. Then, 5 × 10^5^ cells in 300 μL serum-free DMEM supplemented were seeded into the upper part of each chamber, whereas the lower compartments were filled with 500 μl of 10% fetal bovine serum media. Following incubation for 48 hours at 37°C, the insert was incubated in the cell detachment solution. The invasiveness was determined by fluorescence measurement, and the extent of invasion was expressed as an average number of cells per microscopic field.

### Statistical analysis

Statistical analysis was performed with the SPSS15.0 software. The association between expression of STAT1 and survival was analyzed using the Kaplan–Meier’s. The correlation between STAT1 and other clinical parameters was evaluated using Chi square or Student’s t test. A value of p < 0.05 was considered as statistically significant.

## Results

### Expression of STAT1 in esophageal squamous cell carcinoma (ESCC)

**Figure 1:**
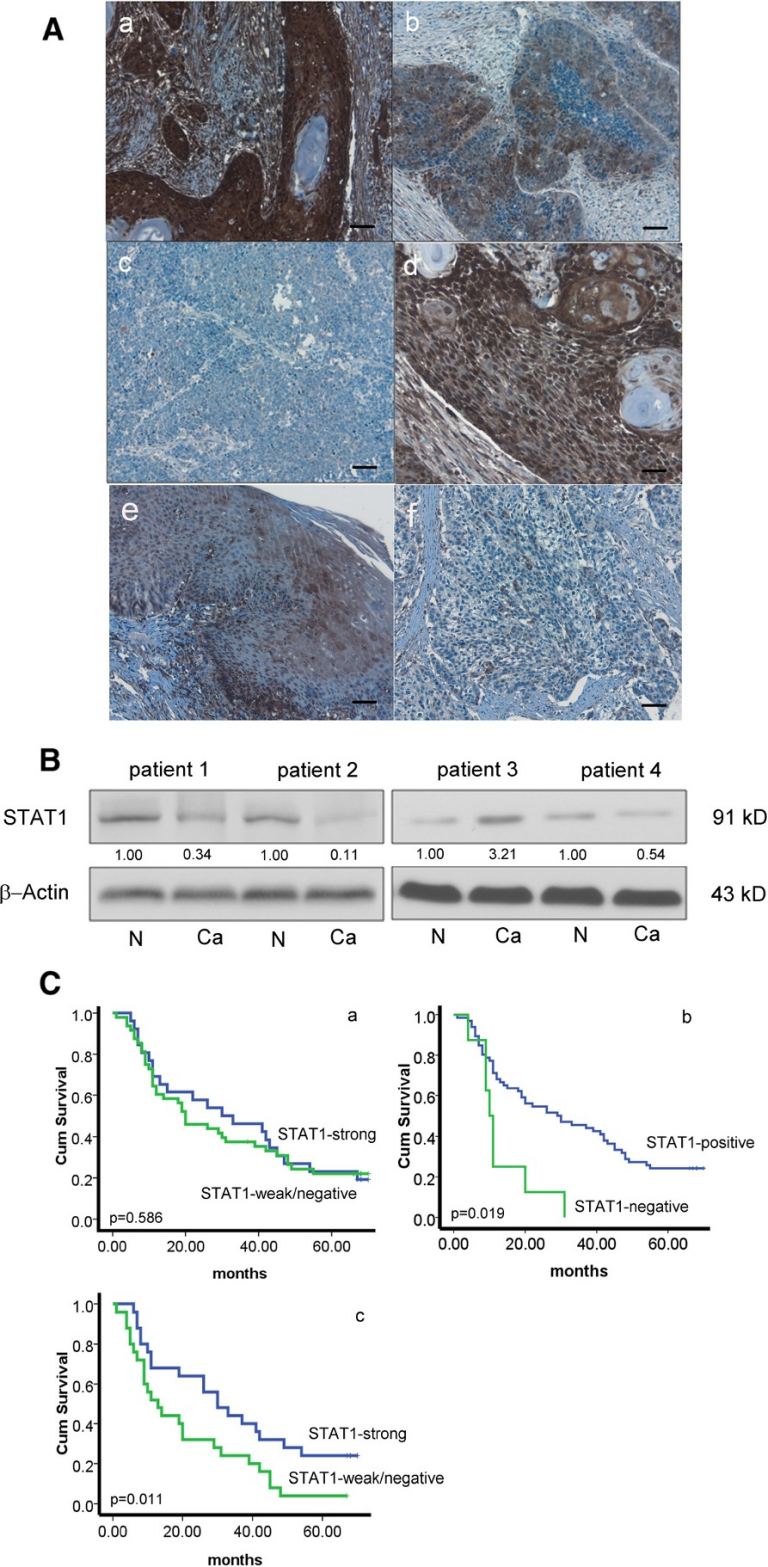
**Heterogeneous STAT1 expression in ESCC. (A)** By immunohistochemistry applied to formalin-fixed paraffin-embedded tissues, variable levels of STAT1 were detectable in most ESCC tumors examined. The staining was predominantly cytoplasmic. Based on the staining intensity, tumors in our cohort was categorized into STAT1-strong **(a)** or STAT1-weak **(b)**; 8 cases were STAT1-negative **(c)** (IHC stain, scale bar, 20 μm). Nuclear staining of STAT1 was detected in some ESCC cases **(d)** (IHC stain, scale bar, 50 μm). The normal epithelium **(e)** from a STAT1-weak tumor (shown in **f**) was also illustrated (scale bar, 20 μm). **(B)** By Western blots, STAT1 expression in ESCC tumors was examined. Compared to the benign esophageal tissue harvested at the surgical margins in the same specimens (labeled as N) cancerous tissues (labeled as Ca) often expressed a lower level of STAT1. Thus, tumors from patient #1, 2 and 4 were categorized as STAT1-low. A small subset of tumors (e.g. that from patient #3) were categorized as STAT1-high, since the expression of STAT1 in the cancerous tissue was appreciably higher than that of the benign esophageal tissues in the same specimen. **(C)** By Kaplan-Meier analysis, we found no significant correlation between overall survival and the expression level of STAT1, when the two groups were defined as STAT1-strong and STAT1-weak/negative **(a)**. In contrast, we found a significant correlation between overall survival and the expression level of STAT1 protein levels when the two groups were defined as STAT1-positive or STAT1-negative **(b)**. With the subset of patients carrying poorly or intermediate-differentiated tumors, those with STAT1-strong tumors survived significantly longer than those with STAT1-weak/negative tumors **(c)**.

To survey the expression of STAT1 expression in our cohort of ESCC, we performed immunohistochemistry (IHC) applied to paraffin-embedded tissues. STAT1 immunoreactivity, assessed based on the presence of cytoplasmic staining, was detectable in the vast majority of cases (123 of 131, 93.8%). The staining intensity was categorized as strong (n = 64, 49.0%) or weak (n = 59, 45.0%) (Figure 1). Of these 123 STAT1-positive tumors, nuclear staining was detectable in 58 (47.2%) cases. The remaining 8 (6.1%) cases had no detectable cytoplasmic or nuclear STAT1 expression. Benign esophageal epithelial cells had relatively strong STAT1 immunostaining in both their nuclei and cytoplasm (illustrated in Figures 1A e and 1A f).

We then validated the IHC findings using Western blots. Of the 131 cases studied by IHC, fresh tumor tissues were available in 57 cases. STAT1 at 91 kd was detectable in all tumors examined, although the intensity was variable, as illustrated in Figure 1B. Densitometry analysis was performed to generate a value for the STAT1 band derived from each of the 57 cases. Based on these values, the 57 cases were categorized as STAT1-high (n = 23, 40.4%) or STAT1-low (n = 34, 59.6%). As shown in Table 1, data generated from the IHC and Western blot studies significantly correlate with each other (p = 0.0003, Fisher exact test). Specifically, 19 (33.3%) cases showing strong IHC for STAT1 were STAT1-high by Western blots; 23 (40.4%) cases showing weak/negative IHC for STAT1 were STAT1-low by western blots.

**Table 1:** **STAT1 expression in ESCC: significant correlation between IHC and Western blot data**

STAT1 expression level by IHC	STAT1 expression level by western-blot			
	High	Low	Total	
Strong	19	11	30	
Weak	4	23	27	
negative	0	0	0	
Total*	23	34	57	P = 0.0003

*all cases in this cohort were positive for STAT1.

### The clinical significance of STAT1 expression in ESCC

We then assessed if STAT1 expression detectable by IHC correlated with various and clinical and pathologic parameters, including gender, location and size of the tumor, lymph node metastasis, histologic grade, depth of tumor invasion and the overall clinical stage. As summarized in Table 2, we found that STAT1 expression inversely correlated with the depth of tumor invasion and tumor size (p = 0.047 and p = 0.029, respectively, Chi square). Cases with strong STAT1 expression also showed a trend toward a higher degree of histologic differentiation (p = 0.074, Chi square). Compared to the poorly differentiated tumors (n = 12), well- or intermediate-differentiated tumors (n = 129) significantly correlated with strong STAT1 immunostaining (p = 0.032, Fisher square). Nuclear expression of STAT1 did not show significant correlation with any of these clinicopathologic parameters.

**Table 2:** **Correlations between STAT1 expression and various clinicopathologic parameters in ESCC**

Parameter		Case number	STAT1 expression by IHC		Result
			Negative/Weak	Strong	
Age	≤57	66	31	35	p = 0.337
	>58	65	36	29	
Gender	Male	98	49	49	p = 0.653
	Female	33	18	15	
Tumor site	Upper	13	8	5	p = 0.572
	Middle	104	51	53	
	lower	14	8	6	
Differentiation	Poor	12	10	2	p = 0.074
	Intermediate	75	37	38	
	Well	44	20	22	
Tumor size	>5 cm	82	48	34	p = 0.029*
	<5 cm	49	19	30	
Depth of invasion	T1-T2	103	48	55	p = 0.047*
	T3-T4	28	19	9	
Lymph metastasis	Yes	68	32	36	p = 0.286
	No	63	35	27	
Clinical stage	1	6	4	2	p = 0.257
	2	55	30	23	
	3	64	32	32	
	4	6	1	5	

*p<0.05.

Clinical follow-up data was available for 74 of the 131 patients included in this study (median follow-up, 31.4 months; range 1-70 months). The survival data was analyzed using Kaplan-Meier’s. Approximately half of these tumors (34 of 74, 45.9%) were assessed strongly positive for STAT1 by IHC, and 40 (54.0%) were assessed weak/negative. The overall survival of patients with STAT1-strong tumors was found to be similar to that of patients with STAT1-weak/negative tumors (35.9 months versus 31.1 months, p > 0.05). In contrast, patients with STAT1-strong/weak tumors (n = 66) had a significantly longer survival compared to those with STAT1-negative tumors (n = 8) (33.6 months versus 13.1 months, p = 0.019). Furthermore, of the 74 patients for whom follow-up data was available, 50 carried poorly or intermediate-differentiated tumors. In this sub-group, patients with STAT1-strong tumors survived significantly longer than those with STAT1-weak/negative tumors (34.6 months versus 20.5 months, p = 0.011). Nuclear STAT1 expression again did not significantly correlate with the overall survival in this sub-group.

### Roles of STAT1 in ESCC cell lines

#### STAT1 expression in ESCC cell lines

**Figure 2:**
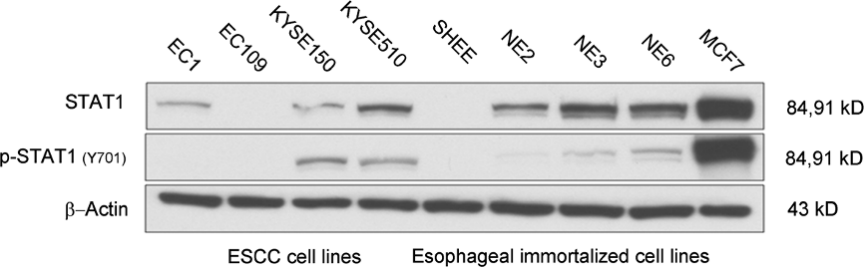
**Expression of STAT1 and phospho-STAT1 in ESCC (n = 4) and esophageal immortalized cell lines (n = 4).** ESCC cell lines included EC1, EC109, KYESE150 and KYSE510 and human esophageal immortalized cell lines included SHEE, NE2, NE3 and NE6. MCF7, a breast cancer cell line, served as a positive control. The expression of STAT1 was heterogeneous among these cell lines, and the expression of phospho-STAT1 was generally in parallel with the expression of STAT1.

In light of the clinical significance of STAT1 in ESCC, we examined its roles in ESCC using an *in-vitro* model. The expression of STAT1 in a cohort of human ESCC cell lines (EC1, EC109, KYSE150 and KYSE510) as well as a cohort of human immortalized esophageal epithelial cell lines (SHEE, NE2, NE3 and NE6) was examined using Western blots. MCF7, an estrogen receptor-positive breast cancer cell line, served as the positive control for STAT1. As shown in Figure 2, we were able to detect STAT1 in 6 of these 8 cell lines; EC109 and SHEE were STAT1-negative. In the 6 STAT1-positive cell lines, EC1 and KYSE150 expressed STAT1 relatively weakly, whereas KYSE510, NE2, NE3 and NE6 expressed STAT1 relatively strongly. The expression of the phosphorylated/activated form of STAT1 (p-STAT1) in these cell lines was also assessed in these 8 cell lines. Except for EC1, all STAT1-positive cell lines expressed p-STAT1, although all of the immortalized cell lines (including NE2, NE3 and NE6) expressed p-STAT1 relatively weakly.

#### The biological impact of STAT1C in ESCC cell lines

**Figure 3:**
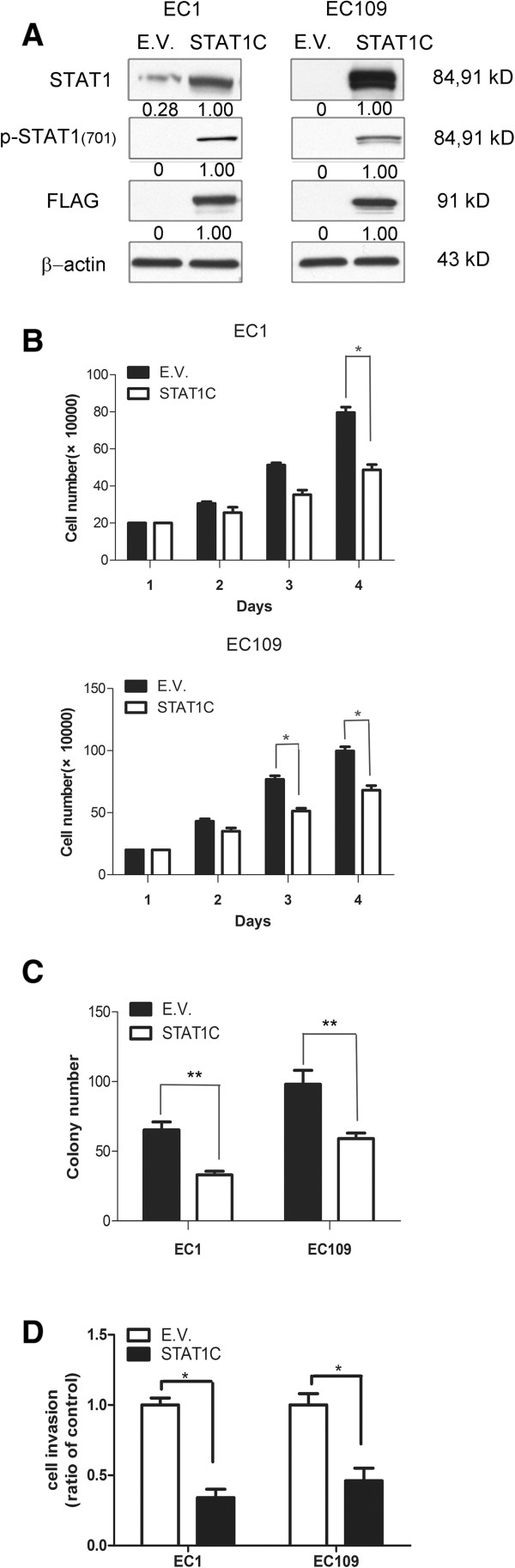
**Gene transfection of** ***STAT1C*** **significantly decreases cell growth and tumorigenecity in ESCC cell lines.** Using Western blot analysis, the gene transfection of *STAT1C* in EC1 and EC109 cells was shown to be effective, since the levels of STAT1, phospho-STAT1 and FLAG were dramatically increased 2 days after *STAT1C* transfection **(A)**. Cell growth, as assessed by trypan blue cell counting, was found to be significantly decreased after *STAT1C* transfection in EC1 and EC109 cells **(B)** (* p < 0.05). Tumorigenecity, assessed by using colony formation assay, was significantly lower in EC1 and EC109 cells transfected with *STAT1C*, as compared to cells transfected with an empty vector **(C)** (** p < 0.001). **(D)** Transwell invasion assay showed that the transfection of STAT1C significantly inhibited cell invasion both ESCC cell lines. Results shown are representative of three independent experiments. (E.V.: empty vector).

**Figure 4:**
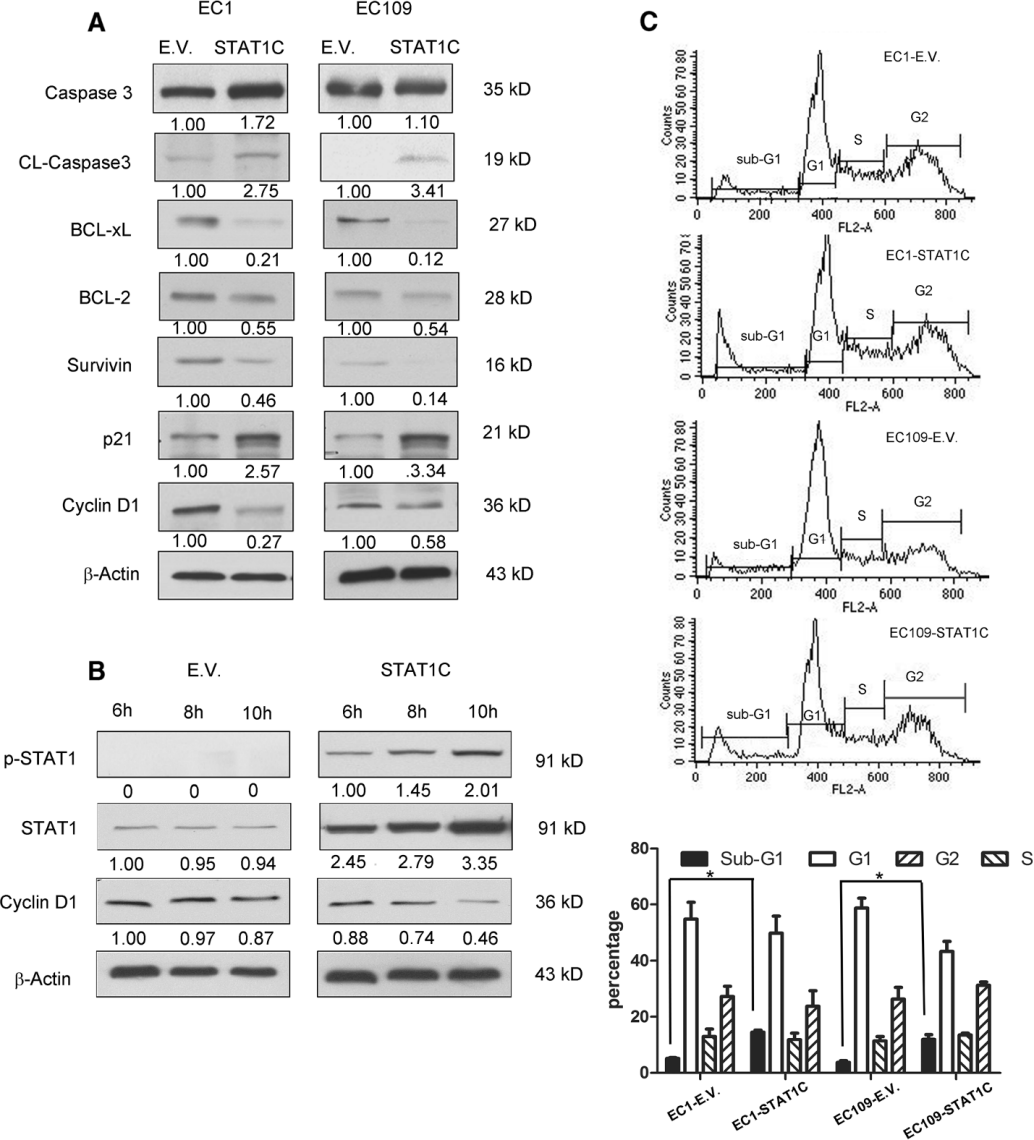
**Gene transfection of** ***STAT1C*** **upregulated apoptosis and induced sub-G**_**1**_ **cell cycle increase.** By western blots, gene transfection of *STAT1C* into ESCC cell lines induced cleavages of caspase 3, downregulated several pro-apoptotic proteins (including BCL-2, BCL-xL, survivin), and promoted G_1_ cell-cycle arrest by decreasing cyclin D1 and increasing p21^waf1^. Cell lysates were collected 2 days after the gene transfection of *STAT1C* in EC1 and EC109 **(A)**. Time course experiments were performed, and the decrease in cyclin D1 expression was detectable as early as 6 hours after *STAT1C* transfection in EC1 cells **(B)**. **(C)** Cell cycle analysis using flow cytometry revealed that STAT1C induced a significant increase in the sub-G1 fraction in both cell lines, EC1 and EC109 (*p < 0.05). All experiments were performed in triplicate, and results from a representative run are shown. (E.V.: empty vector).

Using the ESCC cell lines, we then performed specific *in-vitro* studies. First, we examined the biological impact of enforced expression of the constitutively active form of STAT1 (i.e. *STAT1C*) in ESCC. To this end, EC1 and EC109, both of which were STAT1-weak/negative cell lines, were employed and they were subjected to gene transfection of *STAT1C*. As shown in Figure 3A, the expression of STAT1C was confirmed by the high intensity of the total STAT1 band and the strong expression of FLAG, which was tagged to the *STAT1C* construct. These changes correlated with a significant decrease in the number of viable cells, as assessed using the trypan blue exclusion assay (Figure 3B). As shown in Figure 3C and D, STAT1C transfection in EC1 and EC109 cells led to a significant decrease in colony formation and cell invasion, as compared to cells transfected with the empty vector (p < 0.001 and p < 0.05 in both cell lines). As shown in Figure 4A, the occurrence of apoptosis was supported by the expression of cleaved caspase 3 in both cell lines. Correlating with these changes, there was a marked reduction in the expression levels of several anti-apoptotic proteins including BCL-2, BCL-xL and survivin. Furthermore, we also observed changes in two proteins known to regulate G_1_ cell-cycle progression including p21^Waf1^ and cyclin D1. Specifically, transfection of *STAT1C* into EC1 and EC109 substantially upregulated p21^Waf1^, a negative regulator of G_1_ cell-cycle progression [14]. Cyclin D1, a promoter of G_1_ cell-cycle progression [15], was downregulated. Based on the results of the time-course experiment (Figure 4B), the decrease in the cyclin D1 protein level began as early as 6 hours after *STAT1C* gene transfection, indicating that the decrease in cyclin D1 was not due to the apoptotic activity. As shown in Figure 4C, cell cycle analysis showed a significant increase in the sub-G_1_ fractions in EC1 and EC109 cells transfected with *STAT1C*.

#### The biological impact of siRNA knockdown of STAT1 in ESCC cell lines

**Figure 5:**
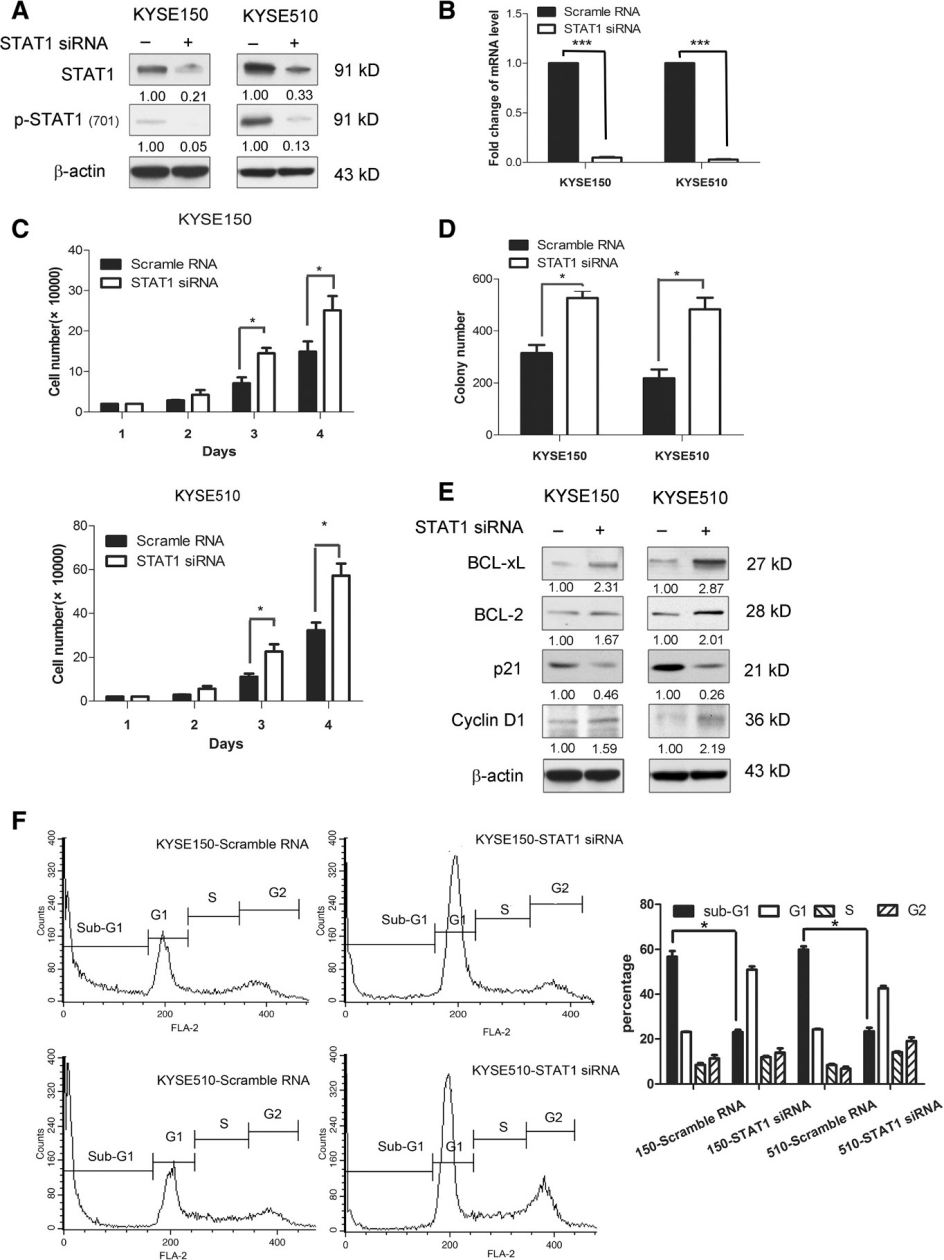
**Inhibition of STAT1 activation by siRNA.** By Western blot analysis, the protein level of STAT1 and phospho-STAT1 were dramatically decreased in KYSE150 and KYSE510 treated with siRNA against STAT1. Cell lysates were collected 2 days after the siRNA transfection **(A)**. The decrease in *STAT1* expression after siRNA treatment was further supported by quantitative RT-PCR (***p < 0.0001) **(B)**. In both KYSE150 and KYSE510, siRNA knockdown of *STAT1* induced a significant decrease in cell growth, assessed by trypan blue eclusion assay. The cell numbers were assessed on day 4 after siRNA transfection. Triplicate experiments were performed and the results of a representative experiment are illustrated (*p < 0.05) **(C)**. Transfection of *STAT1* siRNA into KYSE150 and KYSE510 cells led to a significant reduction in the number of colonies formed, as compared to cells transfected with scrambled siRNA. Triplicate experiments were performed and the results of a representative experiment are shown (***p < 0.0001) **(D)**. By western blots, transfection of *STAT1* siRNA resulted in an appreciable increase in BCL-xL, BCL-2, cyclin D1 and a corresponding decrease in p21^waf1^. Cells treated with scrambled siRNA served as the negative controls. Cell lysates were prepared two days after siRNA transfection **(E)**. Cell cycle analysis using flow cytometry revealed that STAT1 siRNA induced a significant decrease in the sub-G1 fraction in both cell lines, KYSE150 and KYSE510 (*p < 0.05). Results shown are representative of three independent experiments **(F)**.

Next, the biological effects of siRNA knockdown of STAT1 in ESCC cells were evaluated. KYSE150 and KYSE510 cells, which showed the highest level of STAT1 expression among the 8 examined cell lines, were treated with STAT1 siRNA. As shown in Figure 5A and B, STAT1 siRNA induced a dramatic reduction in the STAT1 expression level in both cell lines. With this experimental system, we found that siRNA knockdown of STAT1 significantly decreased the number of viable cells, which was assessed by using the trypan blue exclusion assay (p < 0.05 for both cell lines) (Figure 5C). Furthermore, using colony formation assay, we found that siRNA knockdown of STAT1 of KYSE150 and KYSE510 induced a significant decrease in colony formation (p < 0.0001, p < 0.0001, respectively) (Figure 5D). As shown in Figure 5E, Western blot studies showed changes in the expression of p21^waf1^, cyclin D1, BCL-2 and BCL-xL in a pattern opposite to that seen in EC1 and EC109 cells transfected with *STAT1C*. As shown in Figure 5F, cell cycle analysis showed a significant decrease in the sub-G_1_ fractions in KYSE150 and KYSE510 cells transfected with siRNA against STAT1, compared with the control.

### STAT1 inhibits NF-κB

**Figure 6:**
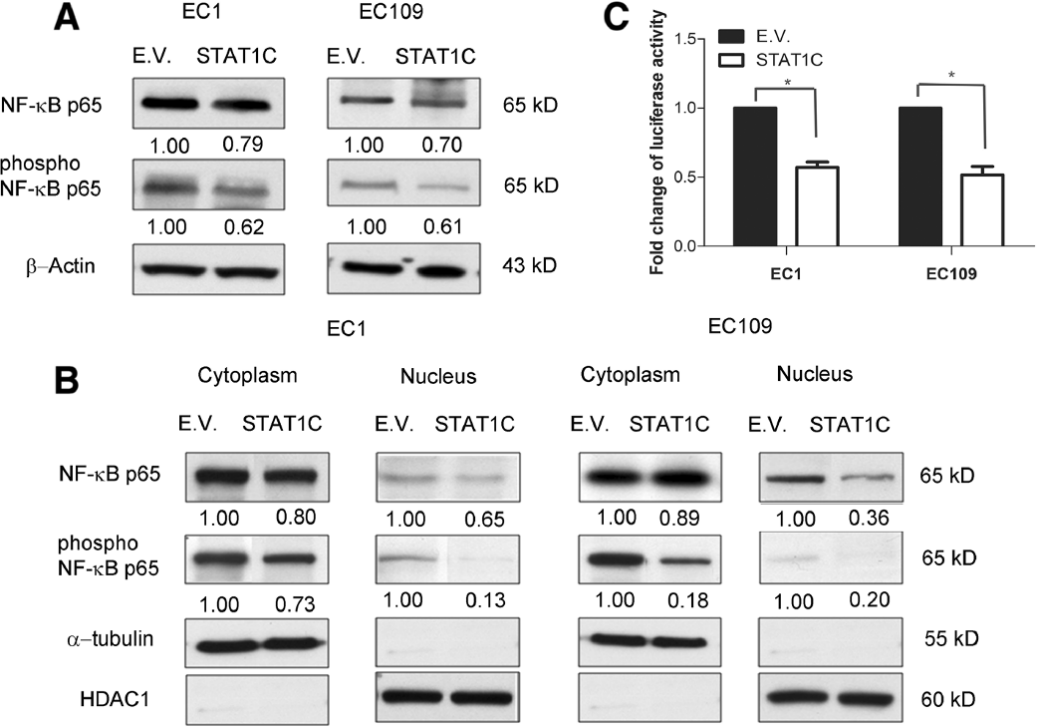
**STAT1C inhibits NF-κB signaling.** Western blot results showed a detectable down-regulation of total p65 and phospho-p65 after *STAT1C* transfection in EC1 and EC109 cells **(A)**. In the same experiment, nuclear/cytoplasmic fractionation studies showed that *STAT1C* induced a substantial decrease in nuclear p65 and phospho-p65 **(B)**. **(C)** Using a NF-κB/luciferase reporter, we found that *STAT1C* gene transfection induce a significant down-regulation of the NF-κB transcription activity in ESCC cells transfected with *STAT1C* cells were harvested 48 hours after the gene transfection. (*p < 0.05) (E.V.: empty vector).

Previous studies have shown that STAT1 can block NF-κB by downregulating TNF-α [16]. In view of the importance of NF-κB in the biology of ESCC [17, 18], we hypothesized that the biological effects of *STAT1C* in ESCC may be mediated by down-regulating the NF-κB signaling. In keeping with this concept, we found that transfection of *STAT1C* into EC1 and EC109 cells resulted in a substantial decrease in the phosphorylation of NF-κB p65, a marker of NF-κB activation [19] (Figure 6A). By subcellular fractionation, we also found that *STAT1C* transfection induced a dramatic decrease in the nuclear localization of NF-κB p65 or phospho-NF-κB p65 in both cell lines (Figure 6B). Lastly, we assessed the transcriptional activity of NF-κB using a commercially available luciferase reporter construct. As shown in Figure 6C, there was a significant down-regulation of NF-κB transcriptional activity after *STAT1C* transfection in both ESCC cells.

### *STAT1C*transfection downregulates STAT3 expression and activation

**Figure 7:**
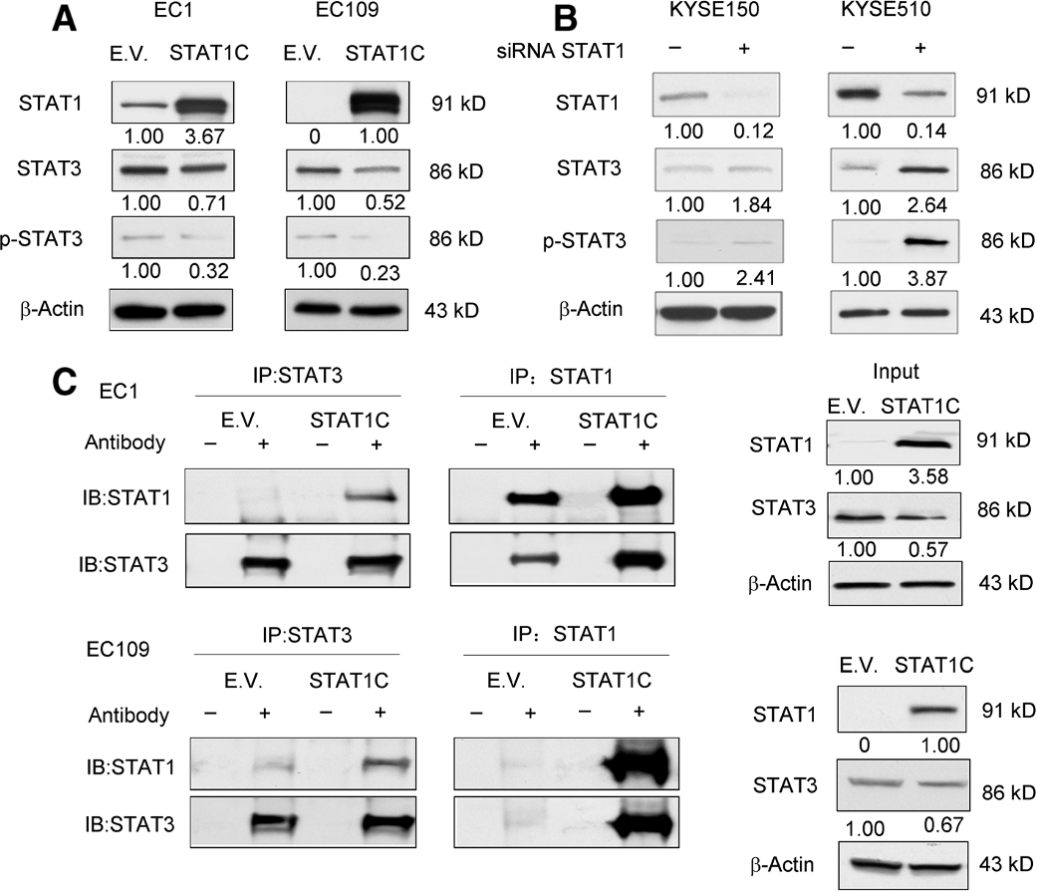
**STAT1C decreases the expression level of STAT3 and phospho-STAT3 and increase STAT1-STAT3 heterodimer formation.** Western blot studies showed that gene transfection of *STAT1C* induced an appreciable decrease in total STAT3 and phospho-STAT3 in EC1 and EC109 cells. Cell lysates were harvested 48 hours after gene transfection **(A)**. In contrast, both STAT3 and phospho-STAT3 were increased in response to siRNA knockdown of STAT1 in KYSE150 and KYSE510 **(B)**. Co-immunoprecipitation experiments revealed that gene transfection of *STAT1C* into EC1 and EC109 induced a substantial increase in STAT1:STAT3 heterodimer; at the same time, the total STAT3 protein level was decreased **(C)**. Cell lysates were prepared 48 hours after gene transfection. (E.V.: empty vector).

Since STAT1 and STAT3 are known to counteract each other during their regulations of various cellular processes [20, 21], we asked if the modulation of STAT1 may have an impact on the expression and/or activation of STAT3. As shown in Figure 7A, we found that the expression levels for STAT3 and p-STAT3 were decreased 48 hours after *STAT1C* transfection into EC109 and EC1 cells. Correlating with these results, siRNA knockdown of STAT1 substantially increased the expression level of STAT3 and p-STAT3 in KYSE150 and KYSE510 (Figure 7A and B). We then assessed how STAT1C transfection might affect the physical interaction between STAT1 and STAT3 using co-immunoprecipitation. As shown in Figure 7C (right panel), by Western blots, transfection of STAT1C again resulted in 30-40% reduction in the expression of STAT3. Co-immunoprecipitation studies (left panel) showed that transfection of STAT1C substantially increased the STAT3-STAT1 binding in both EC1 and EC109 cells. Considering that the total STAT3 protein level was decreased after STAT1C expression, these co-immunoprecipitation data strongly support the concept that the STAT3 homodimers are decreased upon STAT1C transfection.

## Discussion

STAT1 has been reported to regulate cellular differentiation and apoptosis through transcription-dependent as well as transcription-independent mechanisms [22]. There is also evidence that STAT1 carries tumor suppressor functions [23–25]. Decreased or loss of STAT1 expression has been observed in many cancer types such as breast cancer, melanoma and leukemia [2, 26–28]; transfection of *STAT1* or *STAT1C* into cancer cells can arrest their growth by inducing apoptosis and cell-cycle arrest [1, 9, 29]. STAT1 appears to exert its tumor suppressor functions via multiple mechanisms, including downregulation of caspases, Fas, FasL, TRAIL and p21^waf1^ [22]. The apoptosis-inducing function of STAT1 also has been linked to its inhibition of IFN-γ signaling and the subsequent downregulation of several pro-apoptotic proteins including Bak and Bax [2]. Despite the fact that the tumor suppressor function of STAT1 is increasingly recognized, the significance of STAT1 in ESCC has not been clearly defined.

One of the key findings of this study is that the absence of STAT1 expression in ESCC significantly correlates with a worse clinical outcome. Of note, we would like to acknowledge that this observation was based on a comparison between 8 STAT1-negative and 66 STAT1-strong/weak tumors. This highly skewed pattern may have introduced some biases; thus, larger studies using other patient cohorts are needed to confirm this finding. Nonetheless, we do believe that STAT1 holds prognostic value, since we found prognostic significance when we compared STAT1-weak/negative cases and STAT1-strong cases in the cohort of poorly or intermediate-differentiated tumors (Figure 1C). Furthermore, STAT1 expression was also found to correlate with the depth of tumor invasion and tumor size. In this regard, tumor size is one of the determinants of the TNM clinical staging system for ESCC patients [30, 31]. To our knowledge, the prognostic significance of STAT1 in ESCC has never been previously described. Nevertheless, we are aware of a relatively small number of published studies that had found prognostic significance of STAT1 in other types of cancer, such as gastric cancer and melanoma [26, 32].

Correlating with our clinical observations, our *in-vitro* studies have provided further support that STAT1 carries tumor suppressor functions in ESCC. Specifically, we found that transfection of *STAT1C* into ESCC induced apoptosis and cell-cycle arrest, with the effect on apoptosis being more pronounced than that on cell proliferation (Figures 3 and 4). On the other hand, siRNA knock-down of STAT1 in ESCC cells results in opposite effects. In parallel to our findings, we have identified two in-vitro studies in the literature that may have implicated a role of STAT1 in the biology of ESCC [9, 33]. In one of these two studies, IFN-γ and EGF were found to induce apoptosis in KYSE 70 and KYSE 590, two ESCC cell lines [9]. However, while the apoptosis induced by both cytokines was found to correlate with STAT1 activation, whether STAT1 activation is directly responsible for the occurrence of apoptosis in these cells was not clear. In this current study, the use of *STAT1C* and STAT1 siRNA allowed us to pinpoint STAT1 as a key mediator of the induction of apoptosis and cell-cycle arrest in ESCC.

We attempted to delineate the mechanisms by which STAT1 induces apoptosis in ESCC. We found that some of the mechanisms are similar to those previously reported for other cancer types [34]. Thus, gene transfection of *STAT1C* in fibrosarcoma cell lines was found to induce caspase activation and caspase-dependent apoptosis [13]. As we found that STAT1C-induced apoptosis in ESCC correlates with the down-regulation of several pro-survival proteins such as BCL-2 and BCL-xL, previous studies also have shown that STAT1 can promote apoptosis by down-regulating BCL-2 and BCL-xL in multiple myelomas [29].

We also have found evidence that STAT1 can modulate the expression of G_1_ cell-cycle regulatory proteins such as p21^waf1^ and cyclin D1 in ESCC. Upregulation of cyclin D1 has been shown to shorten the G_1_ phase and reported to link to the development and progression of many types of cancer, such as breast cancer [35], gastric cancer [36] and mantle cell lymphoma [37]. In ESCC, cyclin D1 expression detectable by IHC is associated with a worse prognosis [38, 39]. Thus, down-regulation of cyclin D1 induced by STAT1 may partly explain our observation that high STAT1 expression in ESCC is associated with a better clinical outcome. As shown in Figure 4B, our observation that the down-regulation of cyclin D1 occurred as early as 6 hours after *STAT1C* gene transfection suggests that this biochemical change was a consequence of STAT1 up-regulation, rather than a ‘by-product’ of apoptosis.

In various cancer cell types, STAT1 has been previously reported to be an important mediator for NF-κB, which is known to play a critical role in carcinogenesis and chemoresistance in ESCC [40, 41]. The NF-κB signaling pathway was found to be constitutively activated in many ESCC cell lines; down-regulation of p65 has been shown to increase the sensitivity of ESCC cells to chemotherapeutic drugs [17, 18]. With this background, we tested if STAT1 is functionally linked to NF-κB in our experimental model. Our findings that modulation of STAT1 expression changed the expression level of phospho-p65, as well as the nuclear localization of p65/phospho-p65, and these findings support the concept that the STAT1 inhibits the growth of ESCC via its suppression of NF-κB signaling. Similar results have been described in cervical cancer, melanomas and fibrosarcomas [16, 42–44].

STAT3, another member of the STAT family, has been shown to exert opposing biological effects of STAT1 [20, 21, 45]. In contrast with STAT1, STAT3 promotes survival, proliferation and motility of cancer cells, and induces immune tolerance [46]. Some of these opposing biological effects are likely related to the cross-talk between STAT1 and STAT3. For instance, it has been shown that STAT3 can block the activation and function of STAT1 in human monocytic cells [47]. STAT3-knockout mouse embryonic fibroblasts exhibited prolonged IL-6-mediated STAT1 activation and induction of IFN-γ-inducible genes [48]. In this study, we found evidence that STAT1 interferes with STAT3 signaling in ESCC cells using at least two mechanisms. First, STAT1 down-regulates STAT3 as well as phospho-STAT3. To our knowledge, this is a novel finding. Second, co-immunoprecipitation studies showed that *STAT1C* transfection in ESCC cells substantially increased STAT1:STAT3 heterodimer. Since the total STAT3 protein level was decreased after *STAT1C* transfection, it is logical to assume that STAT3:STAT3 homodimers were dramatically decreased at the same time. We believe that these findings are linked to the tumor suppressive effects of STAT1C in ESCC, as previous studies have shown that the relative proportions of STAT1 homodimers, STAT3 homodimers and STAT1:STAT3 heterodimers dictate the cell fate [21, 49, 50]. Based on the literature, other mechanisms where STAT1 inhibits STAT3 signaling may also exist. For instance, it has been shown that STAT1 can compete with STAT3 for the common receptor docking sites or target DNA sequences [2, 20]. Of note, most of these STAT1-mediated effects on STAT3 take place in the cytoplasm, and this may correlate with our observation that cytoplasmic STAT1, rather than nuclear STAT1, was prognostically important.

The role of STAT1 as a tumor suppressor is not without controversy. STAT1 overexpression has been demonstrated in several types of human cancer [51–55]. Some reports also have demonstrated that constitutive STAT1 signaling promotes tumor growth, increases resistance to chemotherapy and radiation, and carries a worse clinical outcome in patients with glioblastoma multiforme [56]. The possible explanations for these rather contradictory findings may be related to the biological heterogeneity of cancer and STAT1 may function as a tumor suppressor or oncoprotein in a cell-type specific manner.

## Conclusion

In conclusion, our findings suggest that STAT1 is a tumor suppressor in ESCC. Loss of STAT1, which is frequent in ESCC, contributes to the pathogenesis of these tumors. We have provided evidence that STAT1 attenuates the tumorigenicity of ESCC by inhibiting the STAT3 and NF-κB signaling pathway, and therefore that activation of STAT1 may be a useful approach to treat ESCC.
